# Early Reinitiation of Obesity Pharmacotherapy Post Laparoscopic Sleeve Gastrectomy in Youth: A Retrospective Cohort Study

**DOI:** 10.1007/s11695-024-07658-8

**Published:** 2025-01-11

**Authors:** Alaina P. Vidmar, My H. Vu, Matthew J. Martin, Aimee G. Kim, Stuart Abel, Madeleine Weitzner, Cynthia E. Muñoz, Ahlee Kim, Kamran Samakar

**Affiliations:** 1 Children’s Hospital Los Angeles and Keck School of Medicine of University of Southern California, Department of Pediatrics, Center for Endocrinology, Diabetes and Metabolism, Los Angeles, USA; 2 Children’s Hospital Los Angeles and The Saban Research Institute Biostatistics Core, Los Angeles, USA; 3 Division of Upper Gastrointestinal and General Surgery, Department of Surgery, Keck Medical Center of University of Southern California, Los Angeles, USA; 4 Children’s Hospital Los Angeles and Keck School of Medicine of University of Southern California, Department of Surgery, Division of Pediatric Surgery, Los Angeles, USA; 5 Children’s Hospital Los Angeles, Department of Psychology, Los Angeles, USA

**Keywords:** Pediatric obesity, Metabolic and bariatric surgery

## Abstract

**Background:**

Bariatric surgery is the most effective intervention for severe pediatric obesity, but a subset of youth experience suboptimal weight loss and/or recurrent weight gain. Early re-initiation of obesity pharmacotherapy postoperatively may improve outcomes, though this has not been evaluated in pediatric populations.

**Methods:**

A retrospective cohort study at a tertiary care children’s hospital evaluated the safety and efficacy of reintroducing obesity pharmacotherapy within six weeks after laparoscopic sleeve gastrectomy (LSG). Youth were offered obesity pharmacotherapy reinitiation at their 2-week postoperative visit. The study compared outcomes between 25 youth who chose early obesity pharmacotherapy reinitiation and 21 who received standard care without restarting medication. Primary outcomes included weight trajectory, eating behaviors, complications, readmissions, and reoperation rates, analyzed using independent t-tests, Chi-squared tests, and logistic regressions.

**Results:**

Between November 2023 and July 2024, 53 youth had surgical consults, and 46 (86% conversion rate; mean age 16.5 ± 1.9 years, mean BMI 53 ± 9.7 kg/m^2^; 70% (32/46) female, 80% (37/46) Hispanic, 87% (40/46) publicly insured) underwent LSG, with 93% (43/46) using obesity pharmacotherapy preoperatively. Mixed-effects multivariate regression, adjusting for baseline BMI, age, and sex, revealed that early reinitiation (5.1 weeks [IQR 3.7, 8.4]) significantly reduced BMI, percent BMI, percent total weight loss (TWL), and percent excess weight loss (EWL) at 3 and 6 months compared to standard care, with no significant differences in complications or readmissions. At 6 months, the mean differences were: %BMI: -6.5% (95% CI: -9.13, -3.86), *p* < 0.001; %TWL: -5.9% (95% CI: -8.52, -3.25), *p* < 0.001; %EWL: Reinitiators: -45.5% vs. standard care: -39.4%; mean difference: -8.2% (95% CI: -14.69, -1.63), *p* < 0.001. Early reinitiation also resulted in a significant reduction in emotional overeating at 3 and 6 months compared to standard care, with mean differences of -2.5 points (95% CI: -3.29, -1.76), *p* < 0.001, and -3.5 points (95% CI: -4.38, -2.69), *p* < 0.001, respectively on self-reported eating behavior questionnaires.

**Conclusion:**

Early obesity pharmacotherapy reinitiation after LSG was safe and well tolerated, improving weight outcomes without negatively impacting complication or readmission rates.

**Supplementary Information:**

The online version contains supplementary material available at 10.1007/s11695-024-07658-8.

## Introduction

Recent projections indicate that by 2050, over half of youth will be affected by obesity, with severe obesity impacting about 7.6% of youth in the U.S [1]. Current treatment approaches for pediatric obesity encompass lifestyle modifications, obesity pharmacotherapy, and metabolic and bariatric surgery (MBS) [2]. Intensive health and behavior lifestyle modification therapy, involving extensive counseling, yields only modest reductions in body mass index (BMI, ~ 3% over 1 year) [3, 4]. Obesity pharmacotherapies combined with lifestyle modification, can achieve more significant body mass index (BMI) reductions (~ 5–17% over 1 year), while MBS, options like Roux-en-Y gastric bypass and laparoscopic sleeve gastrectomy (LSG) offer substantial weight loss (~ 30% over 1 year.) [5, 6]. Given the chronic nature of pediatric obesity, sustained and multifaced approaches are necessary to maintain health benefits over time [2, 4].

Despite its effectiveness, MBS does not guarantee uniform outcomes. Recent ten-year follow-up data from the Teen Longitudinal Assessment of Bariatric Surgery (Teen-LABS) cohort revealed that 11% of participants (28/260) experienced a mean percent change in body mass index of + 7.1%, while 38% of participants (99/260) had a mean percent change of −12.6% at 10 years post-surgery. These findings highlight that a subset of youth experience suboptimal weight loss and recurrent weight gain above their postoperative nadir [7]. Emerging evidence suggests that variations in post-surgical glucagon-like-peptide-1 (GLP-1) levels and increased emotional overeating are linked to suboptimal weight loss outcomes or recurrent weight gain which can reduce or negate any positive impacts on weight-related conditions [8–10]. GLP-1 receptor agonists, such as those approved for pediatric use (liraglutide and semaglutide), and/or other obesity pharmacotherapies, might offer a promising solution as an adjunct to surgery to mitigate these challenges [11–13]. Obesity pharmacotherapies work through multiple mechanisms, to reduce appetite; enhance satiety; and beneficially alter various metabolites to inhibit the reward pathways associated with hedonic eating behaviors which could potentially counteract the counter-regulatory metabolic adaptations that often lead to obesity relapse [14–16].

It is currently common practice to discontinue obesity pharmacotherapy in the perioperative period, and particularly in the early postoperative phase due to concerns about complications related to delayed gastric emptying and gastrointestinal motility. However, there is little available evidence to support these theoretical concerns. The traditional approach of discontinuing obesity pharmacotherapy following surgery may need re-evaluation given the evolving landscape of pediatric obesity treatment [11–13]. Specifically, current protocols generally recommend resuming obesity pharmacotherapy only if initial weight loss is insufficient or if recurrent weight gain occurs within 1–2 years after surgery [12, 13]. However, with increasing use of these medications in youth undergoing bariatric surgery, there is a growing need to explore their optimal use both pre- and post-operatively [4].

There is a critical gap in evidence regarding the use of obesity pharmacotherapy immediately after metabolic and bariatric surgery in pediatric populations [12, 13, 17, 18]. Despite the growing number of pediatric bariatric surgery procedures and the preoperative use of these agents, no standardized protocols exist for their early postoperative use [19–21]. Obesity Pharmacotherapy remain underutilized after surgery, indicating a need to reassess their role and address barriers to their use. To date, no research has examined the efficacy of obesity pharmacotherapy immediately post-surgery in pediatric populations.

At our large tertiary care, safety-net, children’s hospital 570 out of the 1900 youth followed in the comprehensive obesity care program have at least one obesity associated medical problem, and 40% were prescribed an obesity pharmacotherapy. Notably, out of the 50 youth who underwent LSG in the inaugural year of the pediatric bariatric surgery program, 93% were on one or multiple obesity pharmacotherapy at the time of surgery but had stopped postoperatively per standard of care recommendations. However, 62% of these patients reported cravings and increased emotional overeating, despite reduced hunger, prompting many to seek early reinitiation their medication.

Using a shared medical decision-making framework, the multi-disciplinary MBS team, along with patient and caregiver input, developed the PEDIATRIC-RAMP (Pediatric Reinitiation And Management Protocol) to facilitate early obesity pharmacotherapy reinitiation after LSG. This study aims to evaluate the safety, tolerability, and efficacy of the early obesity pharmacotherapy reinitiation protocol in youth with severe obesity who underwent LSG from November 2023 to July of 2024. We hypothesize that early reinitiation of obesity pharmacotherapy, compared to standard care, will be safe, well tolerated, and result in greater weight loss and less self-reported emotional overeating, without negatively affecting nutritional intake or tolerance. These preliminary findings could help bridge the evidence gaps regarding postoperative obesity pharmacotherapy use, potentially guiding future research to improve treatment protocols, enhance weight loss, prevent recurrent weight gain, and optimize long-term cardiometabolic outcomes and eating behaviors in youth.

## Methods

### Study Design

We conducted a retrospective analysis of data from a repository to evaluate outcomes in youth who underwent laparoscopic sleeve gastrectomy at a large tertiary care children’s hospital between November 2023 and July 2024. The study adhered to ethical guidelines for research involving human subjects, with approval from the hospital’s Institutional Review Board. Informed consent was obtained from the youth and one caregiver, and all data were anonymized to ensure participant confidentiality.

## Participants

The study included youth aged 10–21 years who underwent laparoscopic sleeve gastrectomy during the study period and were at least three-months out from their surgery date. Participants were identified from the hospital’s electronic health record and internal program database. Eligibility criteria included: 1) ages 7 to 21 years; 2) BMI > 120% of the 95^th^ percentile; 3) primary surgical weight loss through the pediatric pathway at a tertiary care, safety-net children’s hospital; 4) consent to the clinical data repository. Exclusions applied to those with type 1 diabetes, medications influencing body composition (e.g., prednisone), or syndromes affecting the postoperative course (e.g., Prader Willi Syndrome).

## Group Assignment – Early Anti-Obesity Medication Re-Initiation vs. Standard Care without AOM

At the two-week post-operative visit, all participants completed the Pediatric RAMP checklist (Appendix 1). If meeting hydration, protein, and caloric intake goals without gastrointestinal symptoms, they had the opportunity to restart an obesity pharmacotherapy, guided by a shared decision-making approach. Participants who opted for standard care did not restart any medications. All participants, regardless of obesity medication status, received standard postoperative clinical care, which included clinic visits with the multidisciplinary bariatric team (bariatric surgeon, pediatric endocrinologist, and registered dietitian) at 2 and 6 weeks, and at 3 and 6 months postoperatively.

## Data Collection

Data were extracted from the hospital's obesity-management data repository (REDCap), including electronic health records, surgical logs, follow-up documentation, and self-reported surveys. Collected data encompassed demographic information (age, sex, race, ethnicity, insurance, income), clinical characteristics (BMI, comorbidities, type of surgery, medications, family history), eating behaviors, and post-operative outcomes (weight trajectory, complications, readmissions, reoperations). Additional data on AOM use and adherence were retrieved from the electronic health record.

## Measurements

### Primary Outcome

Anthropometric measures were collected at each clinical visit. Height was measured using a Quick Medical stadiometer (accuracy: 0.1 cm), and weight using the TANITA MC-58030 scale [22] (accuracy: 0.1 kg). BMI was calculated using CDC growth charts [23–27]. Postoperative outcomes included changes in percent BMI, percent total body weight, and percent excess weight (using BMI 8^th^ percentile to define ideal weight) at time of surgical consult, day of surgery, 3-, and 6-months post-surgery.

## Secondary Outcomes

### Safety and Tolerability

Postoperative outcomes were monitored for readmissions and complications documented at 30-, 60-, and 90-days post-surgery. Reoperations and reasons for admissions were systematically recorded by the multidisciplinary bariatric team. Side effects were tracked through weekly phone calls conducted by a study team member to identify and address issues promptly. In these calls patients reported any side effects that occurred since their last clinical encounter, urgent care or emergency room visits, or interactions with a health care clinician in primary or subspecialty care (Appendix 2).

## Self-Reported Eating Behaviors

The Adult Eating Behavior Questionnaire (AEBQ) [28] assessed various eating behaviors. The AEBQ comprises five subscales: Food Responsiveness, Emotional Eating, Satiety Responsiveness, Enjoyment of Food, and Lack of Food Control, evaluated on a 5-point Likert scale. Participants completed the questionnaire online via REDCap during clinic visits, which took approximately 15 min, at surgical consult, 1-,2-, 3-, and 6-months post-operatively. Higher scores indicated a greater tendency toward specific eating behaviors. The AEBQ demonstrated robust psychometric properties, with Cronbach's alpha values ranging from 0.80 to 0.92 [28]. At each clinic visit, youth and one caregiver met with a registered dietitian to assess for any negative compensatory eating behaviors (restricting, purging, binge episodes) collected via semi-structured interview. Additionally, 24-h dietary recalls were collected to assess nutritional intake.

## Statistical Analysis

Baseline demographics and characteristics were summarized descriptively across arms using mean and standard deviation (SD) or median and interquartile range as appropriate for the distribution of continuous variables. Categorical variables are described as a frequency and percentage. Differences in patient characteristics and outcomes between groups were examined using Wilcoxon rank sum test and Fisher's exact test. Additionally, mixed-effects longitudinal regression models (lmerTest package in RStudio) [29, 30] with fixed study arm, study visit, study arm*study visit, and covariates (baseline BMI, age, and sex) were utilized to assess whether there were between-group differences in mean change (from baseline) in clinical outcomes at 3-months and 6-months post-operatively. The study arm * study visit interaction term was included to assess whether the effect of the study arm on clinical outcomes varied over time at different study visits, capturing both the temporal progression of outcomes and the differential effects between treatment arms, while the mixed-effects model accounts for within-subject correlation of repeated measures, allowing for robust inferences despite unbalanced data across time points. Models included random intercepts to account for within-subject correlation between repeated outcomes over time. All participants with data at baseline were included in analyses (*n* = 46). Mixed-effects analyses are generally robust for unbalanced data across study time points where information from the observed data is used to provide information about the missing data without explicitly imputing the missing outcome values using the likelihood-based approach [31]. All results were reported with 95% confidence interval and *p*-value. The statistical significance level was set at 0.05 with two-sided throughout the analyses. All statistical computations were done in RStudio 4.4.1 [32, 33].

## Results

### Participant Characteristics

From the time of protocol implementation, between November 2023 and July 2024, 53 youth had surgical consults, and 46 (86% conversion rate; mean age 16.5 ± 1.9 years, mean BMI 53 ± 9.7 kg/m^2^; 70% (32/46) female, 80% (37/46) Hispanic, 87% (40/46) publicly insured) underwent LSG, with 93% (43/46) using obesity pharmacotherapy preoperatively; Table 1). Over half of the participants (54%, 25/46) self-selected early reinitiation of an obesity pharmacotherapy (median [IQR] time to re-initiation 5.1 weeks ([3.7, 8.4]). The three youth who were not on an obesity pharmacotherapy prior to surgery self-selected the standard of care arm and remained off medication after surgery. Of those who reinitiated an obesity pharmacotherapy, 56% (14/25) resumed their pre-surgery regimen, with 52% (13/25) starting more than one agent. The medications reinitiated postoperatively included semaglutide (17/25), phentermine (11/25), topiramate (7/25), metformin (7/25), and tirzepatide (2/25; Table 2). At surgical consult, and at 1-, 2-, and 3-months postoperatively, there were no missing data points, with a complete dataset available for all 46 youths. At 6 months postoperatively, the following number of youths had not yet reached the time point at the time of publication: Early Reinitiation (*n* = 9) and Standard Care (*n* = 11). Consequently, primary and secondary data were analyzed for the following sample sizes: Early Reinitiators (*n* = 16/25) and Standard Care (*n* = 10/21).

**Table 1:** Clinical and safety outcomes between early obesity pharmacotherapy reinitiators and standard care in a clinical cohort

Patient Characteristic	Total N = 46	Early Reinitiators N = 25	Standard Care N = 21	p^1^
**Demographics**				
Age (years), mean (SD)	16.5 (1.9)	16.8 (2.1)	16.1 (1.7)	0.2
Sex, n (%)				0.2
Male	14 (30%)	10 (40%)	4 (19%)	
Female	32 (70%)	15 (60%)	17 (81%)	
Ethnicity, n (%)				0.3
Hispanic	37 (80%)	22 (88%)	15 (71%)	
Race, n (%)				0.05
Indigenous/First Nations	0 (0%)	0 (0%)	0 (0%)	
Asian	0 (0%)	0 (0%)	0 (0%)	
Black or African	2 (4%)	2 (8%)	0 (0%)	
White	6 (13%)	1 (4.0%)	5 (24%)	
Other	38 (83%)	22 (88%)	16 (76%)	
Insurance, n (%)				0.4
Private	6 (13%)	2 (8%)	4 (19%)	
Public	40 (87%)	23 (92%)	17 (81%)	
**BMI, (kg/m**^**2**^**), mean (SD)**	53 (9.7)	55.4 (9.6)	50.2 (9.3)	0.03
**Obesity Related Complication, n (%)**				
Dyslipidemia	39 (89%)	23 (92%)	16 (84%)	0.6
Anxiety	18 (39%)	11 (44%)	7 (33%)	0.6
Depression	36 (78%)	18 (72%)	18 (86%)	0.3
Obstructive Sleep Apnea	31 (67%)	18 (72%)	13 (62%)	0.5
Pre-Diabetes (HbA1c 5.7–6.4%)	23 (50%)	14 (56%)	9 (43%)	0.6
ALT > 80	9 (43%)	12 (48%)	7 (37%)	0.5
Type 2 Diabetes	14 (30%)	6 (24%)	8 (38%)	0.3
Polycystic Ovarian Syndrome	3 (7%)	0 (0%)	3 (14%)	0.09
**Family History**				0.4
Family History of Obesity	46 (100%)	25 (100%)	21(100%)	
Caregiver Bariatric Surgery^*2*^	43 (98%)	24 (96%)	21 (100%)	> 0.9
**Pre-Op Anti-Obesity Medication, n (%)**				
Phentermine	14 (30%)	11 (44%)	3 (11%)	0.05
Topiramate	13 (28%)	9 (36%)	4 (19%)	0.3
Semaglutide	29 (63%)	17 (68%)	12 (57%)	0.5
Tirzepatide	4 (9%)	2 (7%)	2 (11%)	0.4
Liraglutide	1 (2.2%)	1 (4%)	0 (0%)	0.4
Vyvanse	1 (2.2%)	0 (0%)	1 (4.8%)	0.5
Metformin	12 (26%)	7 (28%)	5 (24%)	0.9
**Treatment timing (months)**				
Start of medical weight management program to surgery consult	5.2 (2.4, 32.4)	10.7 (3.7, 38.9)	4.2 (1.5, 26.2)	0.12
Start of anti-obesity medication to surgery	10.1 (5.1, 25.1)	10.1 (5.2, 31.8)	9.4 (5.2, 17.1)	0.5
Surgical consult to surgery	3.3 (2.7, 4.9)	2.9 (2.7, 3.9)	3.9 (2.9, 5.7)	0.14

1 Wilcoxon rank sum test; Fisher’s Exact test

2 Participants with missing data (*N* = *2*)

**Table 2:** Obesity pharmacotherapy selection pre- and postoperatively in a clinical cohort including mean time to reinitiation after laparoscopic sleeve gastrectomy in those who self-selected early reinitiation (*n* = 25). Over half (56% [14/25]) resumed their pre-surgery regimen, with 52% (13/25) starting more than one agent

n (%)	Pre-Operative Obesity Pharmacotherapy Use	Early Reinitiators' Obesity Pharmacotherapy Selection	Mean Time to Re-Start (Mo. [IQR])
Semaglutide	**18 (67%)**	**18 (67%)**	**1.4 [0.9, 2.9]**
Phentermine	**12 (44%)**	**10 (37%)**	**1.2 [0.9, 3.0]**
Topiramate	**7 (26%)**	**7 (26%)**	**1.1 [0.9, 2.2]**
Metformin	**7 (26%)**	**7 (26%)**	**3 [1.6,6.4]**
Tirzepatide	**2 (7%)**	**2 (7%)**	**1.6 [0.9, 2.0]**
Vyvanse	**1 (4%)**	**0**	**–**
Liraglutide	**1 (4%)**	**0**	**–**

### Change in Weight Trajectory

**Fig. 1:**
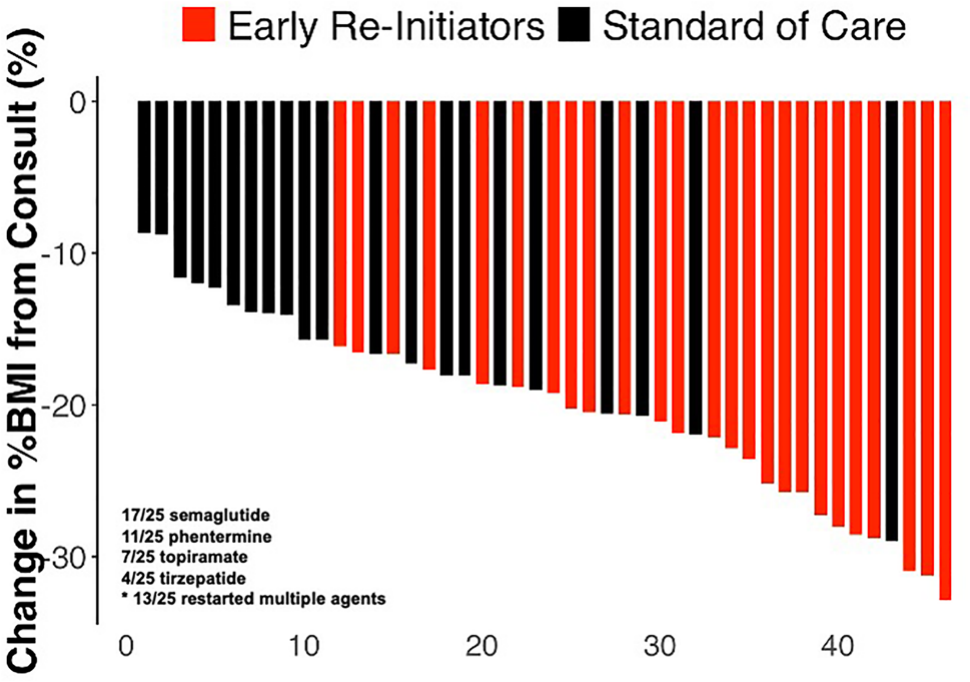
Waterfall plot demonstrating the individual change in percent BMI postoperatively compared to baseline between early obesity pharmacotherapy reinitiators (n = 25) compared to standard care (n = 21) from a clinical sample

Figure 1 demonstrates the within subject change in percent BMI from surgical consult to last anthropometric point available to highlight individual response across the cohort between groups. At 3 months postoperatively, the mean (SD) change from surgical consult in absolute BMI (kg/m^2^) was −10.5 (2.0) kg/m^2^ for early reinitiators and −7.3 (2.3) kg/m^2^ for standard care, representing a difference of −3.2 kg/m^2^ (*p* $\le$ 0.001, Table 2). The correlating mean (SD) percentage change in BMI at this time was −19.4% (4.8%) for early re-initiators and −14.7% (4.4%) for standard care, with a difference of −3 percentage points (*p* < 0.001). At 6 months postoperatively, the mean change in absolute BMI was −13.5 (1.7) kg/m^2^ for early reinitiators (missing data *n* = 9) and −8.9 (1.7) kg/m^2^ for standard care (missing data *n* = 11), showing a difference of −4.6 points (*p* < 0.001). The mean (SD) percentage change in BMI at 6 months was −24.3% (4.7%) for early reinitiators and −18.4% (4.3%) for standard care, with a difference of −5.9% (*p* = 0.004). Additionally, at 3 months postoperatively, youth in the early reinitiation group demonstrated a greater reduction in %TWL and %EWL compared to standard care, representing a difference of −4.4% and 6% respectively (Table 3). Mixed effect multivariate regression analysis, adjusting for baseline BMI, age, and sex, was conducted for each arthrometric measure and revealed that early reinitiation significantly reduced absolute weight (kg), BMI (kg/m^2^), percent BMI, percent TWL, and percent EWL at 3 and 6 months postoperatively compared to standard care (Table 4). At 6 months, mean differences between groups with 95% confidence intervals included: BMI: −4.6 (−5.94, −3.25), *p* < 0.001; %BMI: −6.5 (−9.13, −3.86), *p* < 0.001; %TWL: −5.9 (−8.52, −3.25), *p* < 0.001; %EWL: −8.2 (−14.69, −1.63), *p* < 0.001; Table 4).

**Table 3:** Change in weight trajectory overtime between early obesity pharmacotherapy reinitiators and standard of care in a clinical cohort from initial surgery consult date to 6-months post-operatively

Weight Metric^1^	Total N = 46	Early Reinitiators N = 25	Standard Care N = 21	P^2^
**Weight (kg)**				
** Surgery Date**^**3**^	−4.8 (6.3)	–6.9 (7.3)	–2.3 (3.8)	**0.03**
** 1 month**^**3**^	–14.3 (7.2)	–16.4 (8.4)	–11.7 (4.4)	**0.04**
** 2 months**^**3**^	–20.1 (7.8)	–23.4 (8.0)	–16.2 (5.0)	** < 0.001**
** 3 months**^**3**^	–25.0 (8.5)	–29.1 (7.9)	–20.1 (6.3)	** < 0.001**
** 6 months**^**4**^	–32.8 (9.3)	–37.5 (8.0)	–25.3 (5.5)	** < 0.001**
**BMI (kg/m**^2^**)**				
** Surgery Date**^**3**^	–1.8 (2.0)	–2.5 (2.3)	–0.9 (1.3)	**0.02**
** 1 month**^**3**^	–5.2 (2.4)	–5.8 (2.7)	–4.3 (1.6)	**0.03**
** 2 months**^**3**^	–7.3 (2.4)	–8.4 (2.1)	–5.9 (2.0)	** < 0.001**
** 3 months**^**3**^	–9.0 (2.7)	–10.5 (2.0)	–7.3 (2.3)	** < 0.001**
** 6 months**^**4**^	–11.8 (2.9)	–13.5 (1.7)	–8.9 (1.7)	** < 0.001**
**Percent BMI (%)**				
** Surgery Date**^**3**^	–3.5 (4.3)	–4.9 (4.8)	–1.8 (3.0)	**0.046**
** 1 month**^**3**^	–10.0 (4.8)	–11.2 (5.7)	–8.5 (2.9)	0.14
** 2 months**^**3**^	–13.8 (4.6)	–15.6 (4.8)	–11.7 (3.3)	**0.005**
** 3 months**^**3**^	–17.2 (5.2)	–19.4 (4.8)	–14.7 (4.4)	** < 0.001**
** 6 months**^**4**^	–22.0 (5.3)	–24.3 (4.7)	–18.4 (4.3)	**0.004**
**Percent Total Weight Loss (%)**				
** Surgery Date**^**3**^	–3.3 (9.8)	–4.7 (4.8)	–1.7 (3.1)	0.06
** 1 month**^**3**^	–9.8 (4.8)	–11.0 (5.8)	–8.5 (2.9)	0.2
** 2 months**^**3**^	–13.6 (4.6)	–15.3 (4.9)	–11.6 (3.4)	**0.008**
** 3 months**^**3**^	–17.0 (5.1)	–19.0 (4.8)	–14.6 (4.4)	** < 0.001**
** 6 months**^**4**^	–21.5 (5.3)	–23.6 (4.8)	–18.2 (4.4)	**0.02**
**Percent Excess Weight Loss (%)**				
** Surgery Date**^**3**^	–7.0 (9.8)	–9.9 (10.6)	–3.7 (7.6)	0.067
** 1 month**^**3**^	–20.1 (11.1)	–21.9 (13.3)	–18.0 (7.4)	0.4
** 2 months**^**3**^	–27.7 (11.3)	–30.1 (12.5)	–24.7 (9.2)	0.1
** 3 months**^**3**^	–34.6 (13.6)	–37.3 (13.4)	–31.3 (13.3)	**0.02**
** 6 months**^**4**^	–43.2 (13.0)	–45.5 (12.4)	–39.4 (13.6)	0.2

1 Mean (SD) 2 Wilcoxon rank sum test 3 There are no missing data points for the following time points: surgical consult, 1-,2-, and 3-months post-operatively. 4 At 6-months postoperatively the following youth have not reached the time point postoperatively at time of publication: Total = 20; Early Reinitiation = 9; Standard Care = 11

**Table 4:** Change in weight trajectory overtime between early obesity pharmacotherapy reinitiators and standard of care assessed utilizing a multivariate mixed-effects linear regression model controlling for baseline body mass index, age at time of consult, and sex in a clinical cohort

	**Early Reinitiators (n = 25)**	**p**	**Standard care (n = 21)**	**p**	**Between-group differences**	**p**
	**Mean (95% confidence interval)**		**Mean (95%CI)**		**Mean (95% CI)**	
**Change in Weight (kg) from Surgical Consult**						
3-months^**1**^	–29.1 (−31.3, −26.9)	** < 0.001**	−20.1 (−22.5, −17.6)	< 0.001	−9 (−12.3, −5.7)	** < 0.001**
6-months^**1**^	–37.6 (−40.1, −34.5)	** < 0.001**	−25.4 (−28.5, −22.2)	< 0.001	−12.2 (−16.2, −8.1)	** < 0.001**
**Change in %TWL from Surgical Consult**						
3-months^**1**^	–19 (−20.4, −17.5)	** < 0.001**	−14.6 (−16.1, −13)	< 0.001	−4.4 (−6.5, −2.2)	** < 0.001**
6-months^**1**^	−24.1 (−25.8, −22.4)	** < 0.001**	−18.2 (−20.2, −16.2)	< 0.001	−5.9 (−8.5, −3.2)	** < 0.001**
**Change in BMI (kg/m**^**2**^**) from Surgical Consult**						
3-months^**1**^	–10.5 (−11.2, −9.7)	** < 0.001**	−7.3 (−8.12, −6.5)	< 0.001	−3.2 (−4.2, −2.0)	** < 0.001**
6-months^**1**^	–13.6 (−14.5, −12.7)	** < 0.001**	−9 (−10.0, −8.0)	< 0.001	−4.6 (−5.9, −3.2)	** < 0.001**
**Change in %BMI from Surgical Consult**						
3- months^**1**^	–19.4 (−20.8, −17.9)	** < 0.001**	−14.7 (−16.2, −13.0)	< 0.001	−4.7 (−6.8, −2.5)	** < 0.001**
6-months^**1**^	–24.9 (−26.5, −23.1)	** < 0.001**	−18.4 (−20.4, −16.3)	< 0.001	−6.5 (−9.1, −3.8)	** < 0.001**
**Change in %EWL from Surgical Consult**						
3-months^**1**^	–37.3 (−40.9, −33.7)	** < 0.001**	−31.3 (−35.2, −27.4)	< 0.001	−6 (−11.2, −0.6)	**0.03**
6-months^**1**^	–47.3 (−51.4, −43.1)	** < 0.001**	−39.2 (−44.2, −34.1)	< 0.001	−8.2 (−14.6, −1.6)	**0.01**
**Sub-Analysis: Semaglutide Reinitiators**						
	**Early Semaglutide Reinitiators (n = 17)**	**p**	**Standard care (n = 21)**	**p**	**Between-group differences**	**p**
**Change in Weight (kg) from Surgical Consult**						
3-months^**1**^	−30.3 (−32.9, −27.6)	** < 0.001**	−20.1 (−22.5, −17.6)	< 0.001	−10.2 (−12.3, −5.7)	** < 0.001**
6-months^**1**^	−37.6 (−40.6, −34.5)	** < 0.001**	−25.4 (−28.5, −22.2)	< 0.001	−12.2 (−16.2, −8.1)	** < 0.001**
**Change in %TWL from Surgical Consult**						
3-months^**1**^	−19.7 (−21.5, −17.9)	** < 0.001**	−14.6 (−16.1, −13)	< 0.001	−5.1 (−7.5, −2.8)	** < 0.001**
6-months^**1**^	−24.1 (−26.0, −22.1)	** < 0.001**	−18.2 (−20.2, −16.2)	< 0.001	−5.8 (−8.6, −3.0)	** < 0.001**
**Change in BMI (kg/m**^**2**^**) from Surgical Consult**						
3-months^**1**^	−10.7 (−11.5, −9.7)	** < 0.001**	−7.3 (−8.12, −6.5)	< 0.001	−3.3(−4.5, −2.2)	** < 0.001**
6-months^**1**^	−13.5 (−14.5, −12.7)	** < 0.001**	−9 (−10.0, −8.0)	< 0.001	−4.5(−5.9, −3.2)	** < 0.001**
**Change in %BMI from Surgical Consult**						
3- months^**1**^	−20 (−21.7, −18.2)	** < 0.001**	−14.7 (−16.3, −13.0)	< 0.001	−5.3 (−7.7, −2.9)	** < 0.001**
6-months^**1**^	−24.9 (−26.5, −23.1)	** < 0.001**	−18.4 (−20.4, −16.3)	< 0.001	−6.5 (−9.4, −3.7)	** < 0.001**
**Change in %EWL from Surgical Consult**						
3-months^**1**^	−39.5 (−44.5, −34.9)	** < 0.001**	−31.3 (−35.2, −27.4)	< 0.001	−8.2 (−14.3, −2.1)	**0.01**
6-months^**1**^	−48.4 (−53.4, −43.3)	** < 0.001**	−39.2 (−44.2, −34.1)	< 0.001	−9.2 (−14.6, −1.6)	**0.02**

^**1**^**There are no missing data points at 3-months post-operatively. At 6-months postoperatively the following youth have not reached the time point postoperatively at time of publication: Early Reinitiation = 9; Standard Care = 11**

### Semaglutide Sub-Analysis

**Fig. 2:**
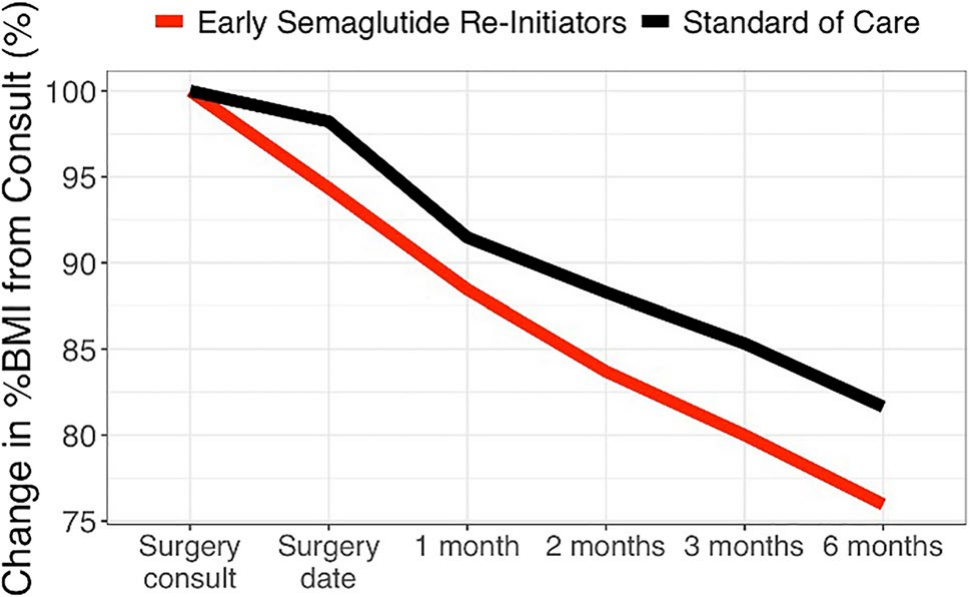
Change in percent body mass index between early semaglutide reinitiators (n = 18) vs. standard care (no obesity pharmacotherapy, n = 21) from a clinical sample

A sub-analysis was conducted on youth who were taking semaglutide 2.4 mg weekly (*n* = 18) for at least 3 months prior to LSG and then reinitiated the medication immediately postoperatively (median [IQR] time to reinitiation 5.0 weeks [3.7, 8.4]. Early reinitiators (*n* = 17) experienced greater reduction in both absolute BMI and %BMI change at 3 months (median [SD[ BMI [kg/m^2^]: −10.7 [2.1] vs. −7.3 [2.3]; p $\le$ 0.001; %BMI: −20 [5.4] vs. −14.7% [4.4]; *p* < 0.001) and mo. 6 (BMI [kg/m^2^]: −13.3 [1.7] vs. −8.9 [1.7]; *p* < 0.001; %BMI: −24% [5.1] vs. −18.4% [4.3]; *p* = 0.02) compared to the standard care (*n* = 21; Fig. 2). Mixed effect multivariate regression analysis, adjusting for baseline BMI, age, and sex, was conducted for each arthrometric measure and revealed that early reinitiation significantly reduced absolute weight (kg), BMI (kg/m^2^), percent BMI, percent TWL, and percent EWL at 3 and 6 months postoperatively compared to standard care (Table 4).

### Eating Behaviors

**Fig. 3:**
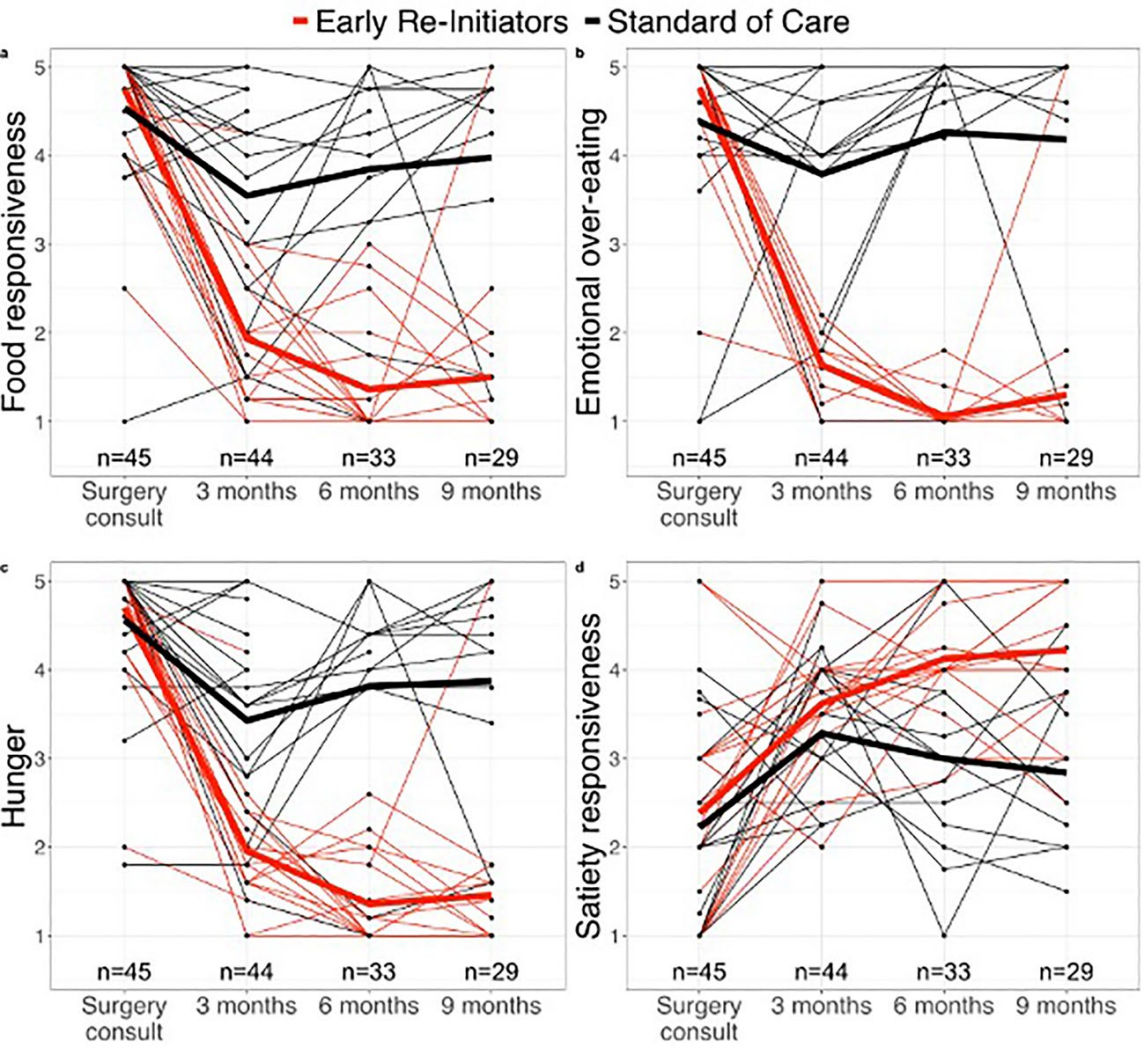
Change in self-reported Adult Eating Behavior Questionnaire scores, completed by the youth participants between early obesity pharmacotherapy reinitiators (n = 25) vs. standard care (no obesity pharmacotherapy, n = 21) from a clinical sample

While all youth (*n* = 46) reported a 50% reduction in hunger on the AEBQ [34] completed 2 and 4 weeks postoperatively, scores for food responsiveness and emotional overeating remained elevated. Compared to standard care, early reinitiators demonstrated a significant reduction in emotional overeating, food avoidance, hunger, food responsiveness and an increase in slowness in eating on the self-reported AEBQ at 3 and 6 months postoperatively (Fig. 3). At 6 months, mean differences between groups with 95% confidence intervals (Early reinitiators: *n* = 19; Standard care: *n* = 11) included: emotional overeating: −4.0 (95% CI: −4.0, −3.9), *p* < 0.001; food responsiveness: −3.4 (−4.0, −2.9), *p* < 0.001; hunger: −3.0 (−4.0, −3.0), *p* < 0.001). Mixed effect multivariate regression analysis, adjusting for baseline BMI, age, and sex, was conducted for each eating behavior sub-category captured on the AEBQ and revealed that early re-initiation significantly reduced food responsiveness, emotional overeating, and hunger at 3 and 6 months postoperatively compared to standard care (Table 5). At 6 months, mean differences between groups with 95% confidence intervals included: emotional overeating: −3.5 (95% CI: −4.38, −2.69), *p* < 0.001; food responsiveness: −2.7 (−3.45, −1.92), *p* < 0.001; hunger: −2.6 (−3.34, −1.87), *p* < 0.001; Table 5). No negative compensatory eating behaviors (restricting, purging, binge episodes) were reported during monthly visits with a registered dietitian. Additionally, 24-h dietary recalls showed that post-operative nutritional intake was adequate for age and weight, meeting all micro- and macronutrient requirements.

**Table 5:** Change in self-reported adult eating behavior questionnaire from baseline at 3 and 6 months postoperatively overtime between early obesity pharmacotherapy reinitiators and standard of care assessed utilizing a multivariate mixed-effects linear regression model controlling for baseline body mass index, age at time of consult, and sex in a clinical cohort

Category of Eating Behavior	Early reinitiators^1^ N = 25	*p*^*2*^	Standard care N = 21	*p*^*2*^	Between-group differences	*p*^*2*^
**Enjoyment in eating**						
**3 months**^**3**^	–0.8 (−1.13, −0.44)	< 0.001	–0.5 (−0.9, −0.17)	0.005	–0.2 (−0.75, 0.26)	0.353
**6 months**^**3**^	–0.5 (−0.86, −0.13)	0.01	–0.2 (−0.68, 0.17)	0.274	–0.2 (−0.8, 0.31)	0.395
**Emotional overeating**						
**3 months**^**3**^	–3.1 (−3.64, −2.59)	< 0.001	–0.6 (−1.15, −0.03)	**0.044**	–2.5 (−3.29, −1.76)	< 0.001
**6 months**^**3**^	–3.7 (−4.22, −3.12)	< 0.001	–0.1 (−0.78, 0.51)	0.718	–3.5 (−4.38, −2.69)	< 0.001
**Food responsiveness**						
**3 months**^**3**^	–2.8 (−3.27, −2.33)	< 0.001	–1 (−1.49, −0.49)	** < 0.001**	–1.8 (−2.5, −1.12)	< 0.001
**6 months**^**3**^	–3.3 (−3.84, −2.85)	< 0.001	–0.6 (−1.24, −0.08)	0.035	–2.7 (−3.45, −1.92)	< 0.001
**Satiety responsiveness**						
**3 months**^**3**^	1.2 (0.74, 1.7)	< 0.001	0.7 (0.14, 1.31)	< 0.001	0.2 (−0.55, 0.86)	< 0.001
**6 months**^**3**^	1.7 (1.2, 2.2)	< 0.001	0.7 (0.14, 1.31)	0.02	1 (0.2, 1.74)	< 0.001
**Hunger**						
**3 months**^**3**^	–2.7 (−3.2, −2.29)	< 0.001	–1.1 (−1.61, −0.65)	< 0.001	–1.6 (−2.27, −0.95)	< 0.001
**6 months**^**3**^	–3.3 (−3.78, −2.83)	< 0.001	–0.7 (−1.26, −0.14)	0.02	–2.6 (−3.34, −1.87)	< 0.001
**Slowness in eating**						
**3 months**^**3**^	2.2 (1.76, 2.68)	< 0.001	1 (0.5, 1.47)	< 0.001	**1.2 (0.57, 1.91)**	0.001
**6 months**^**3**^	2.8 (2.37, 3.32)	< 0.001	0.5 (−0.06, 1.05)	0.09	**2.4 (1.62, 3.09)**	< 0.001

^1^ Median (IQR) ^2^ Wilcoxon rank sum test ^3^ There are no missing data points at 3-months post-operatively. At 6-months postoperatively the following youth have not reached the time point postoperatively at time of publication: Early Reinitiators = 9; Standard Care = 11

### Safety and Tolerability

No significant differences were observed between the early reinitiation and standard care groups in rates of urgent care or emergency room visits (*n* = 4 vs. 3), unplanned readmissions (*n* = 2 vs. 3), or reoperations (*n* = 0 vs. 0) within 30-, 60-, or 90-days post-surgery. In the early reinitiation group, unplanned readmissions occurred for patient 1 (emesis and syncope on post-op day 40 after 1 dose of semaglutide 0.5 mg) and patient 2 (dehydration and poor oral intake intolerance on post-op day 88 after 4 doses of tirzepatide 2.5 mg weekly). In the standard care group, unplanned readmissions occurred for patient 1 (dehydration and poor oral intake intolerance on post-op day 7), patient 2 (constipation and dehydration on post-op day 47), and patient 3 (abdominal pain and cholecystitis on post-op day 95). Urgent care or emergency room visits in the early reinitiation group were for vomiting (post-op day 45, patient on phentermine 15 mg daily), constipation (post-op day 65, patient on metformin 1000 mg twice daily and phentermine 15 mg daily), and headache/vomiting (post-op day 76, patient on semaglutide 2.4 mg weekly). In the standard care group, visits were for vomiting (post-op day 27), constipation (post-op day 46), and nephrolithiasis (post-op day 85).

## Discussion

This study explores a novel approach to enhancing post-surgical outcomes by evaluating the safety, tolerability, and efficacy of early reinitiation of obesity pharmacotherapies following LSG in youth with severe obesity. Current guidelines recommend discontinuing obesity pharmacotherapies postoperatively, but these protocols may not address the complexities of pediatric obesity treatment. Our findings indicate that early reinitiation of obesity pharmacotherapies following LSG is both safe and well-tolerated, with no significant differences in complication rates compared to standard care. Importantly, this approach resulted in a greater reduction in absolute BMI and percent BMI at both 3 and 6 months postoperatively. Additionally, we observed a significant reduction in emotional overeating and other maladaptive eating behaviors in the early reinitiation group, further supporting the potential benefits of this strategy.

These results align with emerging evidence in adult populations supporting the integration of obesity pharmacotherapies into the postoperative care to enhance weight loss and prevent recurrent weight gain [35–38]. Our sub-analysis of semaglutide demonstrated even greater reductions in both absolute BMI and percent BMI compared to standard care. GLP-1 receptor agonists and MBS both modify brain responses to food cues by altering neural connectivity and GLP-1 levels [39–42]. Research indicates that GLP-1 receptor agonists can reduce brain activity in response to food cues, particularly in the insula, and decrease caloric intake and emotional eating even before significant weight loss occurs [41–44]. MBS also improves brain connectivity related to reward based eating drives and reduces cravings for high-calorie foods, effects often observed before substantial weight loss and associated with changes in ghrelin and GLP-1 levels. Incorporating GLP-1 receptor agonists into postoperative protocols could address key factors contributing to suboptimal surgical outcomes [43–45]. These medications may help patients overcome challenges related to weight loss by targeting appetite and satiety mechanisms, thereby potentially enhancing surgical outcomes, and improving long-term health and weight optimization [12, 46].

However, there remains significant variability in the use of GLP-1 receptor agonists across pediatric bariatric programs. A review by Pratt et al. found that only 55% of programs occasionally restarted these medications postoperatively [46]. This inconsistency is likely due to evolving postoperative practices, access barriers, and limited data available in pediatric populations [46]. In the state of California, we were able to conduct this study due to the generally robust public and private insurance coverage for obesity treatment, which is not the case across the country [47, 48]. Social inequities in access to obesity care, including medication treatment, remain a significant barrier, further contributing to the variability in care [4].

The combination of obesity pharmacotherapy, particularly GLP-1 receptor agonists, with MBS in youth raises important cost considerations. GLP-1 receptor agonists, such as semaglutide, typically cost between $800 to $1,200 per month without insurance, which can represent a significant financial burden, especially when considered over the long term [49–53]. In contrast, the cost of MBS, which can range from $15,000 to $30,000 depending on the type of procedure and healthcare setting, is a one-time expense, though it does not account for potential follow-up care, complications, or post-surgical treatments [49, 53]. While the initial cost of combining pharmacotherapy with surgery may appear high, the long-term cost savings could outweigh these expenses. If a multi-modal approach enhances weight loss outcomes and improves obesity-related conditions, it could potentially reduce the need for additional healthcare interventions, such as diabetes management, hypertension treatment, and cardiovascular care, which can subsequently lower healthcare costs over time. In youth, where obesity-related comorbidities often emerge earlier, adding obesity pharmacotherapies to post-surgical care may accelerate the achievement of optimal weight loss, thus improving overall health outcomes and reducing the need for costly long-term treatments. Although the upfront costs are considerable, combining obesity pharmacotherapies with surgery could ultimately lead to cost savings by reducing hospital readmissions and long-term management of obesity-related diseases. However, the economic feasibility of this approach depends on broader access to medications, insurance coverage, and healthcare systems' willingness to prioritize long-term outcomes over initial expenditures. Further research into the cost-effectiveness of combining obesity pharmacotherapies with surgery in pediatric populations is crucial to assess the full economic implications of this integrated treatment approach.

## Limitations

This study has several limitations. First, the retrospective design restricts our ability to establish causal relationships between early reinitiation of obesity pharmacotherapies and improved postoperative outcomes. Reliance on historical data means that while associations can be observed, the absence of randomization and control for confounding variables limits definitive conclusions regarding the efficacy and safety of the early reinitiation protocol. Second, the sample size, while providing preliminary data, may not be representative of the broader pediatric population undergoing LSG or those undergoing Roux-en-Y gastric bypass, as no youths in this cohort underwent the latter procedure. The limited number of participants, particularly those on specific agents like semaglutide, could affect the generalizability of the findings. Additionally, the demographic characteristics of the sample—predominantly Hispanic and publicly insured youth—may introduce selection bias, limiting applicability to more diverse socioeconomic groups. Finally, the short follow-up period of 6 months is a key limitation; the efficacy and safety of early medication reinitiation may evolve over time, highlighting the need for extended follow-up to understand its long-term impact.

## Conclusions

This study underscores the potential benefits of reevaluating post-surgical pharmacotherapy protocols in pediatric obesity management. Early reinitiation of obesity pharmacotherapies after LSG may improve weight management, reduce recurrent weight gain, enhance long-term cardiometabolic outcomes, and improve eating behaviors. Further research is needed to explore the long-term impacts of early obesity pharmacotherapy reinitiation on weight maintenance and comorbidities. We recommend larger multicenter trials to validate these results and investigate how different obesity pharmacotherapies might influence postoperative outcomes.

## Supplementary Information

Below is the link to the electronic supplementary material.

Supplementary file1 (DOCX 17 KB)

Supplementary file2 (DOCX 19 KB)

## Data Availability

The datasets from this study will be available from the corresponding author on written request. The data that support the findings of this study are not publicly available due to their containing information that could compromise the privacy of research participants but are available from the corresponding author [APV] upon reasonable request.

## Abbreviations

AOM: Anti-obesity medications
BMI: Body mass index
zBMI: Body mass index Z-score
LSG: Laparoscopic Sleeve Gastrectomy
%TWL: Percent Total Weight Loss
%EWL: Percent Excess Weight Loss
