# Supplementary material for: Early Reinitiation of Obesity Pharmacotherapy Post Laparoscopic Sleeve Gastrectomy in Youth: A Retrospective Cohort Study
Source: Obes Surg. 2025 Jan 11;35(2):406–18. doi: 10.1007/s11695-024-07658-8 (PMC11835899; doi:10.1007/s11695-024-07658-8)
Supplement: Supplementary file 1 — Supplementary file1 (DOCX 17 KB) [file 11695_2024_7658_MOESM1_ESM.docx]

**Healthcare Utilization:**

1. In the past 2 weeks,
   1. How many times have you seen a doctor or other healthcare professional?
   2. How many times have you gone to the emergency room?
   3. How many times have you gone to the urgent care?
   4. How many times have you called your primary care clinician’s office?
   5. How many times have you utilized any community health resources (e.g., public health programs, local clinics)?
2. When was the last time you visited a healthcare provider for a routine check-up?
3. Do you have a primary care provider (PCP)?

**Side Effects of Obesity Pharmacotherapy:**

1. Have you been prescribed any obesity medications?
2. Which medications for obesity have you taken in the past or are currently taking?
3. How long have you been taking medication(s) for obesity management?
4. Since starting obesity pharmacotherapy, have you experienced any side effects?
5. What specific side effects have you noticed since starting your obesity medication(s)? (e.g., nausea, headache, dizziness, gastrointestinal issues, fatigue, mood changes, etc.)
6. Have these side effects impacted your ability to continue the medication or affected your daily life?
7. Have you had to adjust the dose of your obesity medication due to side effects?
8. Did your healthcare provider discuss possible side effects before prescribing the medication?
9. Have you reported any side effects from your obesity medication to your healthcare provider?
10. If you experienced side effects, did your healthcare provider change your medication or recommend other strategies to manage them?
11. Have you experienced any serious side effects or health complications that you believe may be related to the obesity medication? (e.g., heart palpitations, elevated blood pressure, liver problems, etc.)
12. Since beginning obesity pharmacotherapy, have you experienced any mental health side effects (e.g., anxiety, depression, irritability)?
13. Have you experienced any changes in appetite, eating behavior, or food cravings as a result of the obesity medication?
14. Has your healthcare provider provided advice or treatment to help manage side effects of obesity pharmacotherapy?
