# Supplementary material for: Early Reinitiation of Obesity Pharmacotherapy Post Laparoscopic Sleeve Gastrectomy in Youth: A Retrospective Cohort Study
Source: Obes Surg. 2025 Jan 11;35(2):406–18. doi: 10.1007/s11695-024-07658-8 (PMC11835899; doi:10.1007/s11695-024-07658-8)
Supplement: Supplementary file 2 — Supplementary file2 (DOCX 19 KB) [file 11695_2024_7658_MOESM2_ESM.docx]

**Pediatric-RAMP (Pediatric Reinitiation And Management Protocol)**

Pediatric-RAMP (Pediatric Reinitiation And Management Protocol) promotes a multidisciplinary approach to patient-centered care aimed at facilitating early re-initiation of obesity pharmacotherapy after metabolic bariatric surgery. At the two-week post-operative visit, prior to restarting obesity pharmacotherapy, the following checklist will be used to assess readiness and ensure that the patient is meeting necessary health goals and has no contraindications for resuming medication. The decision to restart obesity pharmacotherapy will be based on the patient's condition, with a shared decision-making approach between the multi-disciplinary team and the patient.

**Hydration, Protein, and Caloric Intake Goals:**

- **Hydration**: Has the patient been meeting their hydration goals?
  - Goal: At least 64 oz (1.9 L) of fluids per day without signs of dehydration (e.g., dry mouth, dark urine, dizziness).
    - Yes
    - No
- **Protein Intake**: Has the patient been able to meet the recommended protein intake goal (e.g., 60-80 grams/day)?
  - Yes
  - No
- **Caloric Intake**: Has the patient been meeting their caloric intake goals?
  - Goal: Typically, 600-800 calories/day in the early post-op phase, as recommended by the healthcare team.
    - Yes
    - No

**Gastrointestinal Symptoms:**

- **No New Gastrointestinal Issues**: Does the patient report no new or unresolved gastrointestinal symptoms (e.g., nausea, vomiting, reflux, bloating, pain)?
  - Yes
  - No
- **Tolerance to Solid Foods**: Has the patient been tolerating soft or solid foods as per the post-operative nutrition guidelines without discomfort or adverse effects?
  - Yes
  - No

**Physical Health and Recovery:**

- **Adequate Healing**: Has the patient shown signs of adequate wound healing and absence of complications (e.g., infection, excessive pain)?
  - Yes
  - No
- **No Active Infections or Complications**: Has the patient been free from any infections or complications since surgery?
  - Yes
  - No
- **Post-Op Weight Loss Progress**: Has the patient experienced appropriate weight loss since surgery, with no signs of severe malnutrition or dehydration?
  - Yes
  - No

**Decision to Restart Obesity Pharmacotherapy:**

- **Shared Decision-Making Discussion**: Has a thorough discussion occurred between the healthcare clinician and patient regarding the option to restart obesity pharmacotherapy, including benefits and risks?
  - Yes
  - No
- **Participant Decision to Restart Obesity Pharmacotherapy**: Has the patient expressed a clear desire to restart obesity pharmacotherapy based on informed consent?
  - Yes
  - No

**Screening for Contraindications:**

- **No Contraindications to Obesity Pharmacotherapy**: Are there any contraindications for restarting obesity pharmacotherapy, such as severe dehydration, active gastrointestinal issues, or other post-surgical complications?
  - Yes
  - No
  - If "Yes," please specify: _____________________________
- **Mental Health Considerations**: Has the participant been evaluated for any psychiatric conditions prior to surgery that should be considered with the use of certain obesity pharmacotherapy?
  - Yes
  - No
  - If "Yes," results: _____________________________

**Final Evaluation:**

- **Restart Obesity Pharmacotherapy as Per Patient's Choice**: Is the patient ready and eligible to restart obesity pharmacotherapy based on the above criteria?
  - Yes
  - No
- **Alternative Plan if Obesity Pharmacotherapy is Not Restarted**: If the decision is made to not restart obesity pharmacotherapy, has an alternative management plan been discussed (e.g., behavioral interventions, nutrition counseling)?
  - Yes
  - No

**Healthcare Clinician's Notes:**

- **Next Steps for Care**:

This checklist will help guide clinical decision-making for restarting obesity pharmacotherapy after metabolic and bariatric surgery while ensuring the safety and well-being of the patient in the post-operative period.
